# Label-Free Biosensing with High Selectivity in Complex Media using Microtoroidal Optical Resonators

**DOI:** 10.1038/srep13173

**Published:** 2015-08-14

**Authors:** Erol Ozgur, Pelin Toren, Ozan Aktas, Ersin Huseyinoglu, Mehmet Bayindir

**Affiliations:** 1 UNAM-National Nanotechnology Research Center, Bilkent University, 06800 Ankara, Turkey; 2 Institute of Materials Science and Nanotechnology, Bilkent University, 06800 Ankara, Turkey; 3 Department of Physics, Bilkent University, 06800 Ankara, Turkey

## Abstract

Although label-free biosensors comprised of optical microcavities inherently possess the capability of resolving molecular interactions at individual level, this extreme sensitivity restricts their convenience for large scale applications by inducing vulnerability towards non-specific interactions that readily occur within complex media. Therefore, the use of optical microresonators for biosensing is mostly limited within strictly defined laboratory conditions, instead of field applications as early detection of cancer markers in blood, or identification of contamination in food. Here, we propose a novel surface modification strategy suitable for but not limited to optical microresonator based biosensors, enabling highly selective biosensing with considerable sensitivity as well. Using a robust, silane-based surface coating which is simultaneously protein resistant and bioconjugable, we demonstrate that it becomes possible to perform biosensing within complex media, without compromising the sensitivity or reliability of the measurement. Functionalized microtoroids are successfully shown to resist nonspecific interactions, while simultaneously being used as sensitive biological sensors. This strategy could pave the way for important applications in terms of extending the use of state-of-the-art biosensors for solving problems similar to the aforementioned.

Label-free detection of chemical and biological species is an indispensable analytical method providing fast and reliable qualitative and quantitative information regarding the composition of analyte, and comprehension of interaction among biomolecules^1^,^2^,^3^. Among various label free biodetection techniques such as surface plasmon resonance^4^ and quartz crystal microbalance^5^, optical microresonators of whispering gallery mode (WGM) type, where light is confined in a circular path inside the periphery of the resonator by means of continuous total internal reflection^6^, particularly possess outstanding potentials by virtue of their extreme sensitivity towards the alterations of the refractive index of the media they reside within^7^. Light can be evanescently coupled to these optical microcavities using tapered optical fibres as resonant modes^8^, which can be tracked by continuously scanning the wavelength of a tuneable laser around the resonant wavelength and observed as Lorentzian shaped dips in the transmission spectrum of the tapered fibre^9^,^10^. The linewidth of the resonant mode is inversely proportional to the quality factor (*Q*) of the microresonator, representing its ability of storing optical power^11^. Especially surface tension induced microcavities, which surface roughness, one of the main sources of optical losses due to scattering from the surface, is diminished by a thermal treatment causing the melting and reflowing of the material constituting the microresonator^12^, such as microspheres^13^ and microtoroids^14^, have extremely narrow resonance linewidths. This enables detection of even single adsorption events causing shifts in the resonance wavelength^15^. There are several important examples of microresonator optical biosensors based on the WGM shift principle for detection of particular biological species such as DNA^16^, viruses^17^, and proteins^18^.

Although this supreme sensitivity of the WGM microresonators is initially conceived as an advantage, there is a major concern regarding the lack of intrinsic discrimination mechanism among different species inducing refractive index alterations through non-specific interactions between the microresonator and the constituents of its surrounding media, except for the measurements taken in precisely determined experimental conditions, such as in buffer solutions. This represents a conundrum regarding the applicability and reliability of optical microresonators with high selectivity in real life situations such as specific detection of biomolecules like cancer markers in serum samples. Several works have been demonstrated the applicability of the WGM microresonators as biosensors for measurements in complex media, where an initial calibration is performed with using the media in order to subtract the signal caused by non-specific molecular interactions^18^,^19^; however, the sensitivity of the biosensors compared to the measurements in buffer solutions were significantly decreased in each case, probably due to steric effect of the weak yet abundant non-specific interactions caused by irreversible adsorption of constituents, mostly proteins, from the media onto the resonator surface. Utilization of well-established silane based anti-fouling surface modification^20^ could significantly reduce the effect of non-specific interactions in biosensing with the silica based WGM microresonators, as demonstrated previously^21^; however, functionalization of these biosensors with molecular probes, such as antibodies, without compromising their protein resistant characteristics remains as an essential challenge. The protein resistance of molecular coatings heavily depend on their functional groups^22^; therefore, having a coating with various functionalities, such as protein resistance and bioconjugability, is challenging to obtain in terms of efficiency. Besides, unlike the thiol based coatings, where mixing compounds with different characteristics is straightforward^23^, due to the complex nature of silane chemistry^20^, forming multifunctional coatings with silane based molecules, convenient for optical microresonators, is a meticulous endeavour.

Recently, we have suggested that coating of an organosilane molecule having methylphosphonate functional group could be used to form a simultaneously protein resistant and bioconjugable silica surface^24^. In this article, we demonstrate, for the first time, that this multifunctional coating could indeed solve the aforementioned problem of biosensing with the WGM microresonators by enabling recognition of antigens after functionalization of microtoroids with corresponding antibodies with high sensitivity in complex media and also by suppressing non-specific interactions simultaneously. Figure 1 schematically describes our approach in this work. The microtoroids functionalized with the described robust process being done mostly at room temperature, which is applicable to many other types of the silica based WGM microresonators, are shown to possess a substantial protein resistance, while this does not deteriorate their biosensing capabilities. The outcomes of this research could considerably facilitate the utilization of the WGM microresonators as optical biosensors with extreme sensitivities in much challenging tasks including point of care diagnostics, food safety and public health, and civil defence against bioterrorism or biological weapons.

Microtoroids were fabricated in a similar fashion with one of our previous works^25^, by applying photolithography, wet and dry etch, and CO_2_ laser reflow sequentially to silicon wafers having 2 μm thermal oxide layer on top (Fig. 2a–d, see Methods for details). Light from a tuneable laser operating at 1550 nm is coupled into the microtoroids using tapered optical fibres formed by simultaneously heating a fibre with a hydrogen torch and pulling it towards opposite directions by two reciprocal linear motion stages at constant pulling speed (0.10 mm/s). A closed-loop piezo controlled 3-axis stage is used during optical coupling, which is monitored by two optical microscopes. Light intensity is continuously measured with a powermeter, and the data from the laser output wavelength and powermeter is captured with an oscilloscope (Fig. 2e). All the equipment during fibre tapering and optical coupling were controlled by custom developed software. Figure S.1 shows the images of microtoroids evanescently coupled with tapered fibres (Fig. S.1a–c), as well as the data of mode structure of microtoroids, obtained by scanning the laser wavelength, depending on the free spectral range and polarization of the incoming light (Fig. S.1d and S.1e), and spectrum of a single resonance with its corresponding Lorentzian fit (Fig. S.1f). The *Q* measured here is 3.95 × 10^6^. Microtoroids were first coated with 3-(Trihydroxysilyl) propyl methylphosphonate (THPMP), which forms an anti-fouling layer over silica surfaces, using a slightly modified recipe than we used in our previous research^24^ (Methods). Piranha cleaned and THPMP coated microtoroids were compared regarding non-specific adsorption of albumin–fluorescein isothiocyanate conjugate (FITC-BSA) by confocal microscopy. As could be seen from Fig. 3, there is a considerable non-specific protein adsorption over Piranha cleaned microtoroid, while the THPMP coated microtoroid exhibits a significant protein resistant characteristic. After THPMP coating, the microtoroids were covalently functionalized with antibodies against human interleukin 2 (α-IL-2) (Methods). For biosensing experiments, we placed the microtoroid over a poly(methyl methacrylate) (Plexiglas) substrate having inlet and outlet streams (1 mm in diameter) for fluidic flow. Following optical coupling with a tapered fibre, buffer solution was introduced, covering both the microtoroid and the inlet/outlet streams. Then, a glass slide was placed over the Plexiglas platform entrapping the measurement solution to form a stable microaquarium (Fig. S.2). At a constant inlet/outlet flow rate, infusion and withdrawing of the analyte were done through the microaquarium system with using two individual syringe pumps. Mathematically modelling the microaquarium system, and assuming the system volume (*V*) as constant and homogenous, unsteady state solution for concentration of the analyte within the system (since the mass Fourier number <<1 in such a case) was performed. The concentration change inside the microaquarium with respect to time could be expressed as:


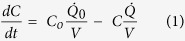


Where *C* is the concentration (mol/mm^3^) of analyte inside the microaquarium at a given time *t*, *C*_*o*_ is the concentration (mol/mm^3^) of the infused analyte solution, *V* is the volume (mm^3^) of the microaquarium, and 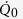, 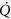 are input and output volumetric flow rates (mm^3^/min), respectively. Noting that 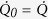, the solution of Eq. (1) under proper boundary conditions is:


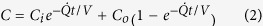


Since *C*_*i*_, the initial analyte concentration within the microaquarium, equals to zero, the Eq. (2) can be simplified to:


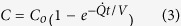


Which the change of analyte concentration within the microaquarium with respect to time, can be calculated.

The effect of complex media directly applied over the bare and the functionalized microtoroids was interrogated. As buffer solution and complex media, 1× Phosphate Buffered Saline (PBS) and 10% (v/v) Fetal Bovine Serum (FBS) in 1× PBS were used, respectively, in order to reduce the effect of viscosity. As could be seen from Fig. 4a considerable reduction in the WGM resonance shift was observed from the functionalized microtoroid as compared to the bare one. The resonance shift with respect to FBS concentration, within the microaquarium, is also calculated according to Eq. (3), and given in Fig. 4b. It could be observed that especially at lower FBS concentrations, the reduction in the WGM resonance shift against FBS is more significant, and it is more than 50% at 1% in PBS. After this concentration, the resonance shift tends to be saturated in both bare and functionalized microtoroid. The shift caused by FBS infusion occurs cumulatively by the refractive index change (bulk effect) and nonspecific protein adsorption (surface effect). Although the change in the refractive index in the environment affects both bare and functionalized microtoroids, the adsorption of proteins on the microtoroid surface appears to be eliminated significantly after functionalization.

In order to investigate the biosensing capabilities of functionalized microtoroid, we measured the sensitivity in PBS buffer. Human interleukin 2 (IL-2) was used as an analyte, with a concentration of 16 nM, in 1× PBS. Figure 5 represents the temporal response of the resonance wavelength after analyte infusion in 1× PBS, and also the information related to the effect of IL-2 concentration on the WGM shift. The limit of detection (LOD) of functionalized microtoroid in buffer was calculated as 0.10 nM.

Finally, we demonstrated the applicability of biosensing within complex media, by performing IL-2 detection measurements in 10% FBS in PBS (Fig. 6). The experiments in complex media were initiated in 10% (v/v) FBS rather than 1× PBS, for avoiding a WGM shift would have been caused due to ambient refractive index change, and only observing the effect of IL-2 on the resonance wavelength. IL-2 protein was also diluted in 10% FBS. Mouse IL-12 protein, which does not have an affinity towards α-IL-2, was used as a control. Comparing responses of functionalized microtoroids towards IL-2 and IL-12 in 10% (v/v) FBS, a significant WGM shift was observed from the analyte solution containing IL-2 (Fig. 6 red line), while there was no significant response towards IL-12 (Fig. 6 green line). The LOD of the THPMP coated microtoroid in 10% (v/v) FBS is calculated as 0.11 nM, suggesting that the microtoroidal biosensor substantially preserved its sensitivity in complex media.

## Discussion

Silica based optical microresonators are heavily susceptible to non-specific adsorption of proteins^26^. Therefore, a myriad of strategies were developed to suppress this undesirable phenomenon^27^,^28^. Optical resonator based biosensors particularly suffer from this adverse effect of non-specific interactions, observed as a considerable decrease in their sensitivities^18^,^19^ during measurements in complex media. 10% (v/v) FBS in 1× PBS is used in this research as complex media, with total protein concentration is in average 3.8 mg/ml in its diluted form^29^, of which 2.3 mg/ml are albumins that non-specific adsorption behaviours onto unmodified silica surfaces have been recently investigated by our group^24^. Our sensitivity, on the other hand, corresponds to 1.7 ng/ml, which is a considerably lower amount compared to that of all other proteins in the media. Although there are previous studies in which 75%^30^ or undiluted serum^19^ were used, we believe 10% (v/v) FBS in 1× PBS could still be considered as a highly complex media, as previously utilized several times in different studies^18^,^31^,^32^. In terms of anti-fouling characteristics, THPMP coating of microresonators showed a comparable, or even slightly better protein resistance than a previous work^21^ when the results of FBS induced resonance wavelength shift are considered. Being bioconjugable, on the other hand, is a crucial advantage of use of the THPMP in microresonator functionalization for biosensing purposes, and we were able to perform biosensing in complex media without our sensitivity being compromised.

The goal accomplishment of our research is demonstration of a novel surface functionalization suitable for all silicon based optical microcavities. One of the main advantages of this technique is its robustness. The surface functionalization strategy is not only convenient for optical microresonators, but also for different silicon based biosensors such as QCM. Besides, if the silane moieties could be replaced with thiols, our surface modification scheme could also be applied to various sensors having Au/Ag coated surfaces, such as SPR biosensing measurements.

The microresonators were treated with Piranha solution in mild conditions in order to activate the surface prior to THPMP coating. Although it was reported previously that Piranha solution could hamper the quality factor (*Q*) by forming micro-cracks on the resonator surface^33^, we did not observe such an effect on the *Q* values obtained during this study (Fig. S.3).

The sensitivity of optical microresonators as biosensors depends on their *Q* values, which is correlated with the optical loss. In air or vacuum, one of the main sources of optical losses is scattering from the surface, which could significantly be reduced in surface tension induced microcavities^12^. *Q* values of higher than 10^9^ for microspheres^34^ and 10^8^ for microtoroids^14^ were previously reported. In this aspect, obtained *Q* values are at least one order of magnitude lower than the expected value. On the other hand, another important source of optical losses is due to absorption by adsorbed water molecules on the microresonator surface especially in infrared wavelengths, reducing the *Q* gradually after the fabrication of the microcavity^12^. Since we performed the experiments in aqueous media, the effect of water as an enlargement on the full width half minimum (FWHM) of the resonance wavelength (Fig. S.4) was observed directly. This reduction is consistent with previous reports in the literature, and could be compensated with use of a near infrared visible laser^17^.

The LOD of the measurements were calculated by considering standard deviation (*StDev*) before infusing the analyte into the system, and the LOD value was considered as 3 *x* *StDev* (Methods). Minimum detectable IL-2 concentration with our system is approximately 50 times higher than the commercial ELISA kits^35^, yet this is partially due to the maturity in cytokine detection with ELISA^36^, while the detection limit of ELISA for other proteins, such as cancer markers^19^, could be much higher, where our system becomes favourable, not only in terms of sensitivity, but also for the time required to do the measurement, where it is possible to complete the process within half an hour, while it takes more than four hours with a standard ELISA measurement. Also, the LOD value we calculated is quite high compared to other research^18^, where 0.3 fM of IL-2 was detected in 10% (v/v) FBS, while we were able to detect five orders of magnitude higher concentrations under same circumstances. This shows that our system has a considerably higher LOD compared to the microtoroids modified with another surface modification strategy, physisorption; yet, this difference solely depends on the wavelength of the tuneable laser utilized, because in the described work a laser centred at 681.5 nm was used, forming much narrower resonance linewidths, while we used a laser at 1550 nm, and our *Q* was limited with absorption of water. On the other hand, our limit of detection compared to the total protein concentration is quite high, showing a substantial selectivity, while the selectivity of the aforementioned surface was not mentioned.

A difficulty one encounters when working with surface tension induced optical resonators is how to integrate the optical coupling system with microfluidics. Since tapered fibres are used in optical coupling, putting all the system in a microfluidic setup is a challenging task. Forming a microaquarium is an inadequate approach, since the microaquarium should be formed by micropipetting, at least, in our experimental setup. This, especially, causes a change in the concentration of FBS between the initial liquid drop and the infused analyte, probably due to adsorption of some serum constituent on the walls of the syringes and the tubes. In the absence of a humidity chamber as in our setup, evaporation losses might also contribute to the concentration change with respect to time, however, putting together the setup within a humidity chamber with good isolation is also a tedious task. These two factors together cause a small but significant shift on the resonance wavelength towards the opposite direction, which can be observed in the response of microtoroid when IL-12 in 10% (v/v) FBS was infused. On the other hand, we were able to detect the presence of IL-2 with the functionalized microtoroid under the same circumstances, and the sensitivity was not seriously affected in complex media. This experiment could be improved by using a third syringe pump which 10% (v/v) FBS would be infused until equilibrium was reached, but in overall, our data is adequate for confirming our hypothesis regarding use of simultaneously bioconjugable and protein resistant surface chemistry in optical resonator based biodetection.

In summary, we successfully demonstrated the application of the multifunctional silane coating in biosensing with optical microresonators for the first time. We achieved biodetection with high sensitivity in complex media, and our sensitivity was not affected by the presence of serum constituents. This proof-of-principle demonstration of using a multifunctional surface modification represents the importance of having rigorously controlled biomolecular interactions on the biosensor surface, and the technique we describe here could find important applications in biosensing with optical microresonators, and might extend their use from precisely determined laboratory conditions towards real life problems in broader fields of healthcare, food and agriculture, and defence, where biosensing simultaneously with high selectivity and sensitivity is essential.

## Methods

### Microtoroid fabrication

Silicon wafers with 2 μm thermal oxide layer were spin coated with AZ4533 photoresist, and circular pads were formed by photolithography and subsequent developing. The oxide layer was removed with buffered oxide etch (BOE), and the silicon below the silica disks were etched isotropically using sulphur hexafluoride (SF_6_) plasma in an inductively coupled plasma device. After the removal of the residual photoresist using O_2_ plasma, the microdisks were reflowed with a CO_2_ laser.

### Optical coupling

SMF28 optical fibres were tapered with a hydrogen torch while simultaneously being pulled towards opposite directions with two reciprocal linear motion stages. Microtoroids were coupled to the light from an external cavity laser operating around 1550 nm via the tapered fibre, using a closed-loop 3-axis piezo controller. The wavelength of the laser was continuously swept around the resonant wavelength. The setup was controlled and the data were captured via custom built software. The resonant wavelength of each data frame was determined by performing Lorentzian fit as a post process analysis.

### Surface modification

Microtoroid surface was cleaned with mild Hellmanex solution (1% v/v), ethanol, acetone and dH_2_O, respectively. Then, Piranha solution (H_2_SO_4_:H_2_O_2_ 3:1 v/v) at 60 °C was used for microtoroid surface activation. Piranha solution is highly reactive and should be handled with care in a fume hood. Then, the activated microtoroids were coated with 2.5% (v/v) THPMP in MeOH solution containing 5% (v/v) dH_2_O (at pH 4.6 adjusted with acetic acid) for 1 hour at RT. After gently washing with the MeOH solution, the THPMP coated microtoroids were then cured at 100 °C in vacuum for 1 hour. Anti IL-2 antibody (Sigma, GW22461F) was covalently conjugated to the THPMP coated microtoroid surfaces after incubation in 5 mM EDC in the MES buffer (50 mM MES, 0.1 mM NaCl in dH_2_O at pH 6.0 adjusted with NaOH) for 2 hours at RT. To remove residual EDC molecules, the surfaces were washed with the MES buffer and 1× PBS rapidly, and then soaked into 2.5 μg/ml anti IL-2 solution in 1× PBS at 4 °C for 2 hours on a lab shaker. The Anti IL-2 conjugated microtoroids were washed with 1× PBS to remove unbound Anti IL-2 and stored in 1× PBS at 4 °C.

### Biosensing experiments

Analyte infusion and withdrawal were performed using two syringe pumps operating at infusion/withdrawal rates of 5 μl/min or 10 μl/min, depending on the experiment. Human IL-2 antigen (Sigma, SRP3085) was diluted to a final concentration of 250 ng/ml in 1× PBS, or 1× PBS containing 0.1 × FBS. After the optical coupling condition was satisfied, initial solution was dropped over the microtoroids by the help of a micropipette, and the solution was covered with a microscope slide forming a microaquarium around the microtoroid. The resonance wavelengths were investigated in under-coupled regime in order to minimize the linewidth of the coupled mode. Baseline was measured for 5 min before the simultaneous initiation of infusion and withdrawal. After 10 minutes of the liquid flow, the data were captured for 5 more minutes to observe the effect of stopping the infusion/withdrawal. As a control, mouse IL-12 antibody (R&D Systems, 419-ML) was used.

### Data analysis

The resonance wavelength of each measurement was determined by applying Lorentzian fit as a post process analysis. The cumulative data were smoothed by adjacent averaging, considering an averaging time of 1 min. The LOD was determined after calculation of standard deviation within a time frame of 1 min before the infusion.

### Confocal microscopy

Confocal imaging was performed using 20× objectives. Samples were incubated in FITC-BSA (1 mg/ml) for 1 h prior to measurement. Argon laser at 488 nm was used for excitation, and emission over 505 nm was collected using photomultiplier tubes. The settings for gain and offset were kept constant throughout all measurements. The pinhole was set to 3 airy units, and the images were formed by averaging each scan line 16 times.

## Additional Information

**How to cite this article**: Ozgur, E. *et al.* Label-Free Biosensing with High Selectivity in Complex Media using Microtoroidal Optical Resonators. *Sci. Rep.* **5**, 13173; doi: 10.1038/srep13173 (2015).

## Supplementary Material

Supplementary Information

## Figures and Tables

**Figure 1:**
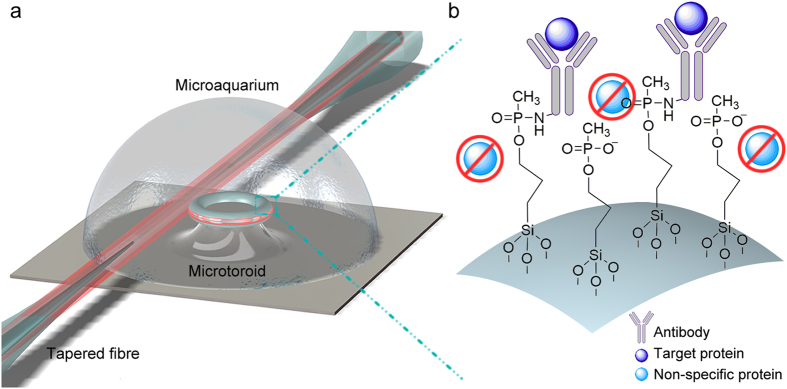
Selective biodetection using microtoroids. Microtoroidal optical resonator modified with silane molecules was used to attain a label-free biosensor with high selectivity. (**a**) Biosensing experiments were performed in liquid media using a microtoroidal optical resonator. Light is evanescently coupled to the microtoroid using a tapered optical fibre. (**b**) The surface chemistry used throughout this study depends on attaching covalently 3-(Trihydroxysilyl) propyl methylphosphonate (THPMP) molecules onto the microtoroid surface. The THPMP forms a protein resistant thin film, as well as convenient sites for bioconjugation of molecular probes such as antibodies via activation of the THPMP coating with N-(3-Dimethylaminopropyl)-N′-ethylcarbodiimide hydrochloride (EDC). This method enables selective detection of biological species in complex media.

**Figure 2:**
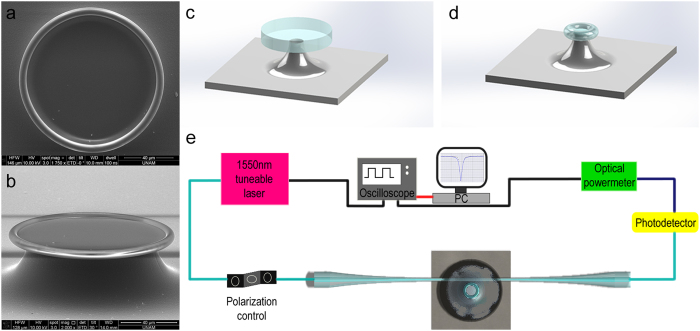
Microtoroid fabrication and optical coupling setup. Microtoroids (**a**,**b**) were fabricated by reflow of microdisks following microfabrication (**c**) by a CO_2_ laser (**d**). The optical setup consists of a tuneable laser, a polarization controller, a powermeter and an oscilloscope. All setup could be controlled with a custom built software.

**Figure 3:**
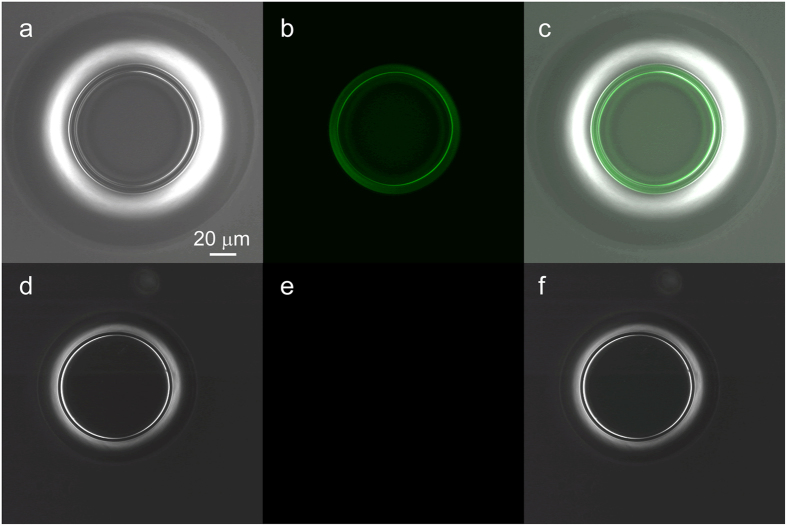
Demonstration of protein resistant characteristics of THPMP coated microtoroids. Confocal microscopy is used in order to demonstrate the effectiveness of THPMP coating over microtoroids in terms of protein resistance. Piranha-treated and THPMP coated microtoroids were incubated in solution of FITC-BSA conjugate (1 mg/ml) in 1× PBS buffer for 1 h prior to confocal imaging. (**a**–**c**) DIC, fluorescence and merged images of Piranha treated microtoroids incubated in FITC-BSA conjugate solution. (**d–f**) DIC, fluorescence and merged images of THPMP coated microtoroids incubated in FITC-BSA conjugate solution. All measurement were taken under the same microscope and illumination configurations. Virtually no fluorescence is observed from the THPMP coated microtoroids.

**Figure 4:**
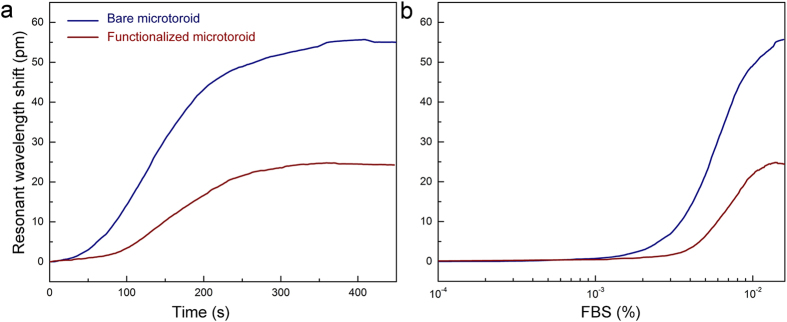
Comparison of responses of functionalized and bare microtoroids towards complex media. After the microtoroids were functionalized with THPMP and antibodies, their responses in terms of WGM resonant shift were compared with bare microtoroids towards complex media. (**a**) Temporal increase of WGM resonant wavelength after infusion of 10% (v/v) FBS with an infusion rate of 5 μl/s beginning at *t* = 0. The infusion continued for 300 s, and the bare microtoroid (blue) showed a higher sensitivity compared to functionalized microtoroid (red). (**b**) WGM resonance shift with respect to FBS concentration.

**Figure 5:**
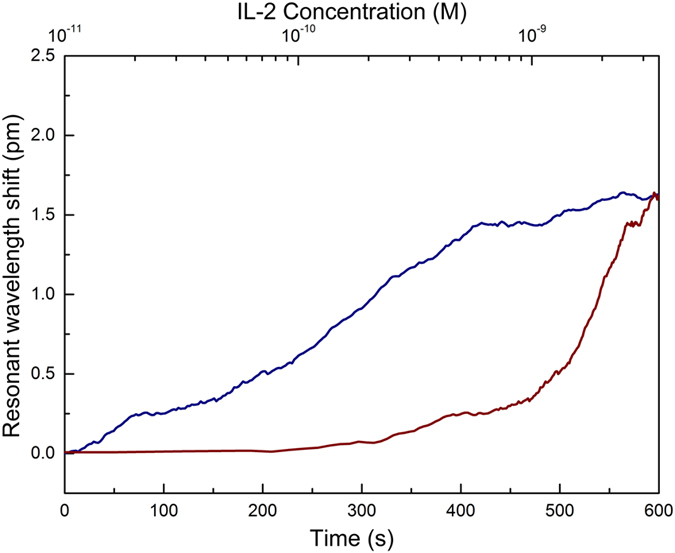
Sensitivity of the microtoroid biosensor. Human IL-2 protein in 1× PBS buffer was used as an analyte for α-IL 2 antibodies. The infusion started at t = 0 and continued for 600 s with an infusion rate of 5 μl/s. The blue line represents the shift with respect to time, while the red line is the response in terms of WGM resonance shift with respect to IL-2 concentration.

**Figure 6:**
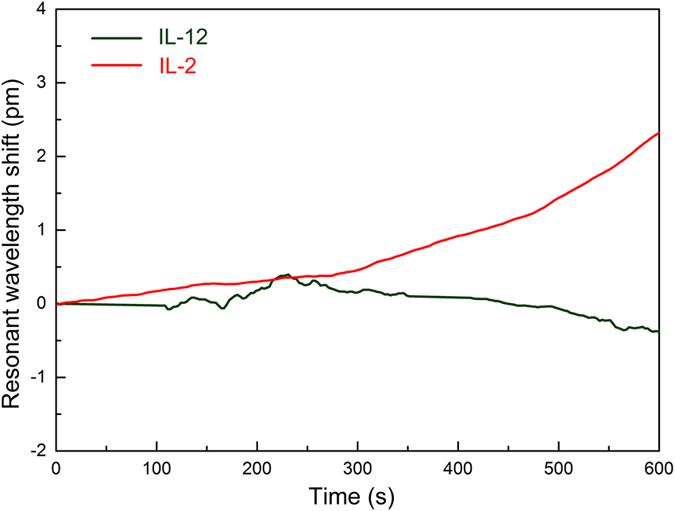
Selective biodetection in complex media. Human IL-2 protein was detected in 10% (v/v) FBS in 1× PBS buffer. As a control, mouse IL-12 proteins were used instead as an analyte in complex media. The infusions started at t = 0 with an infusion rate of 10 μl/s, and the infusion continued for 600 s. The functionalized microtoroids showed a significant WGM resonance shift after IL-2 infusion in complex media (red), while a similar shift could not be observed with IL-12 (green).
