# Supplementary material for: Label-Free Biosensing with High Selectivity in Complex Media using Microtoroidal Optical Resonators
Source: Sci Rep. 2015 Aug 14;5:13173. doi: 10.1038/srep13173 (PMC4642504; doi:10.1038/srep13173)
Supplement: Supplementary Information [file srep13173-s1.pdf]

Supplementary Information

Label-Free Biosensing with High Selectivity in Complex Media Using Microtoroidal Optical Resonators

Erol Ozgur[1], Pelin Toren[1,2], Ozan Aktas[1], Ersin Huseyinoglu[1,2] & Mehmet Bayindir[1,2,3*]

[1]UNAM-National Nanotechnology Research Center, Bilkent University, 06800 Ankara, Turkey

[2]Institute of Materials Science and Nanotechnology, Bilkent University, 06800 Ankara, Turkey

[3]Department of Physics, Bilkent University, 06800 Ankara, Turkey

[*]E-mail: bayindir@nano.org.tr

Table of Contents

S1 Optical coupling of light into the microtoroids 3

S2 Biological measurement setup 4

S3 Effect of Piranha treatment on microtoroid quality factor (Q) 5

S4 WGM resonances in aqueous environment 6

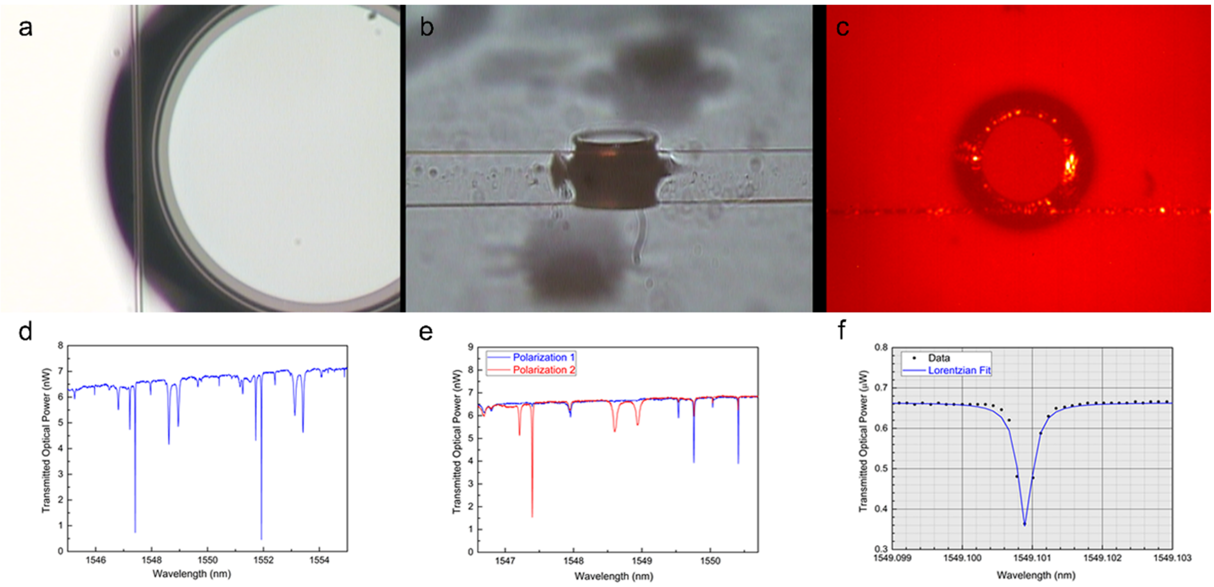

Figure S1 | Optical coupling of light into the microtoroids. Light is evanescently coupled into the microtoroids using tapered optical fibres. The coupling was simultaneously monitored using two cameras from top (a) and side (b) of the microtoroids. Coupling of infrared light could also be directly observed using an IR camera (c). Different orders of coupled optical modes could be observed simultaneously (d), where these modes could be separated as TE (red) or TM (blue) (e). High resolution scanning of individual modes and subsequent fitting into a Lorentzian enables the estimation of Q values of the microtoroids (f).

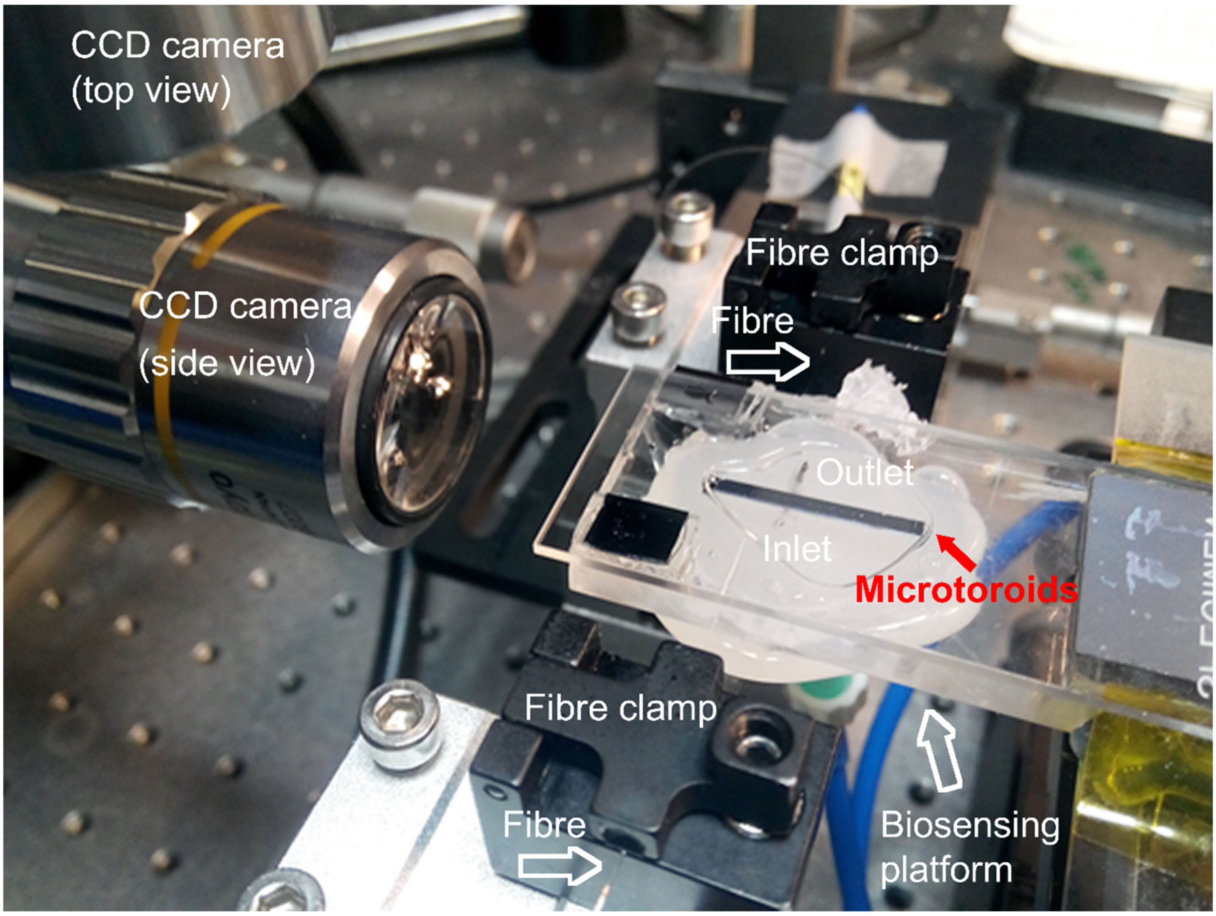

Figure S2 | Biological measurement setup. The system is constructed over a platform of Plexiglas with two drill holes for simultaneous liquid influx and withdrawal. A microaquarium is formed by placing a microscope slide over the liquid droplet in which the measurement takes place. In order to keep the microaquarium volume constant, infusion and withdrawal was performed with equal flow rates. Optical coupling is performed in the microaquarium, and visualized by the camera at the top.

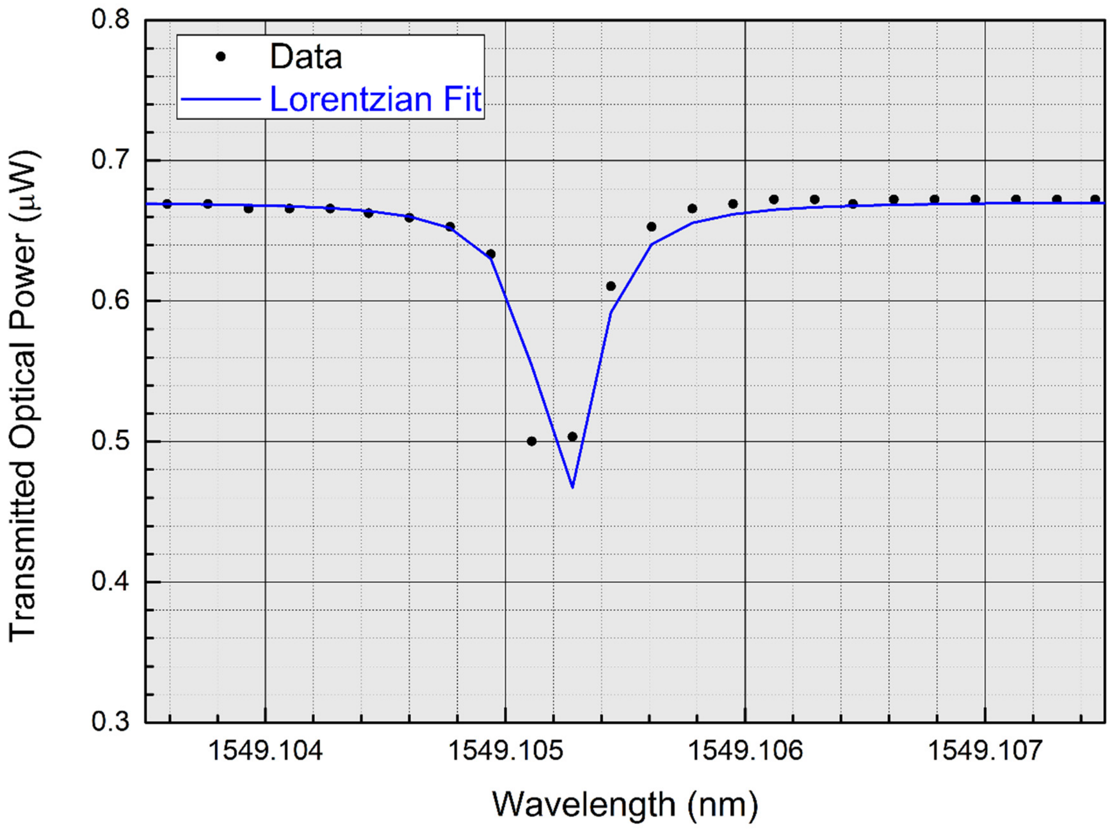

Figure S3 | Effect of Piranha treatment on microtoroid quality factor (Q). The microtoroid was treated with Piranha solution at 60 °C for 30 min. No significant decrease in the quality factor was observed.

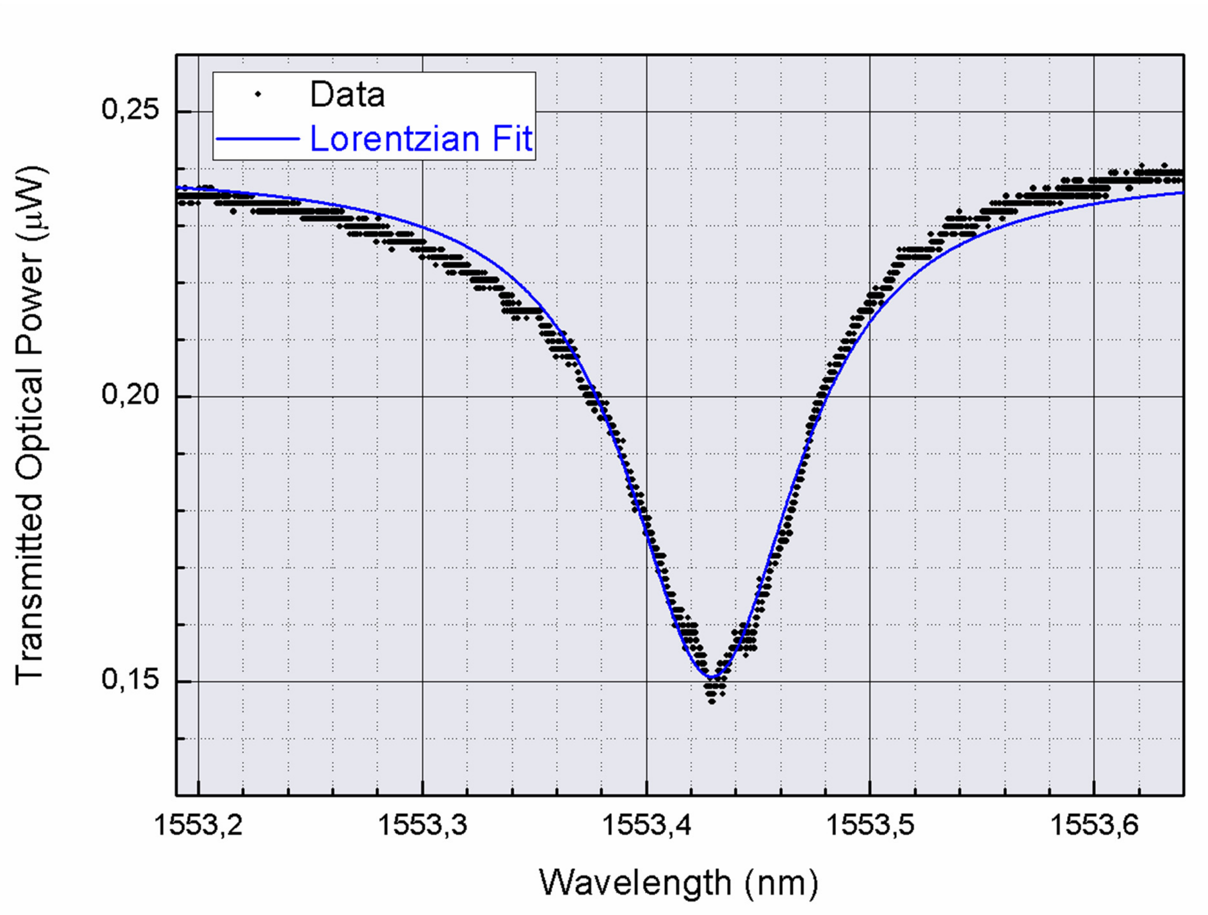

Figure S4 | WGM resonances in aqueous environment. Since light travels in the circumference of the microtoroid, it interacts with the water molecule in the media as well. Since there is some absorption of water at 1550 nm, this causes a broadening in the linewidth of the resonant mode, which corresponds to a decrease in Q value, thus a reduction in the sensitivity of the measurement.
